# Effects of Plyometric Jump Training on Measures of Physical Fitness and Sport-Specific Performance of Water Sports Athletes: A Systematic Review with Meta-analysis

**DOI:** 10.1186/s40798-022-00502-2

**Published:** 2022-08-29

**Authors:** Rodrigo Ramirez-Campillo, Alejandro Perez-Castilla, Rohit K. Thapa, José Afonso, Filipe Manuel Clemente, Juan C. Colado, Eduardo Saéz de Villarreal, Helmi Chaabene

**Affiliations:** 1 Exercise and Rehabilitation Sciences Laboratory, School of Physical Therapy, Faculty of Rehabilitation Sciences, Universidad Andres Bello, Santiago, Chile; 2 Department of Physical Education and Sport, Faculty of Sport Sciences, University of Granada, 18011 Granada, Spain; 3 School of Physical Education and Sports, Rashtriya Raksha University, Gandhinagar, 382305 India; 4 Centre for Research, Education, Innovation, and Intervention in Sport (CIFI2D), Faculty of Sport of the University of Porto, Rua Dr. Plácido Costa, 91, 4200-450 Porto, Portugal; 5 Escola Superior Desporto e Lazer, Instituto Politécnico de Viana do Castelo, Rua Escola Industrial e Comercial de Nun’Álvares, 4900-347 Viana do Castelo, Portugal; 6 Research Center in Sports Performance, Recreation, Innovation and Technology (SPRINT), Melgaço, Portugal; 7 Instituto de Telecomunicações, Delegação da Covilhã, 1049-001 Lisbon, Portugal; 8 Research Group in Prevention and Health in Exercise and Sport (PHES), University of Valencia, Valencia, Spain; 9 Physical Performance Sports Research Center (PPSRC), Universidad Pablo de Olavide, Seville, Spain; 10 Department of Sports and Health Sciences, Faculty of Human Sciences, University of Potsdam, 14469 Potsdam, Germany

**Keywords:** Plyometric exercise, Musculoskeletal and neural physiological phenomena, Human physical conditioning, Movement, Muscle strength, Resistance training

## Abstract

**Background:**

A growing body of literature is available regarding the effects of plyometric jump training (PJT) on measures of physical fitness (PF) and sport-specific performance (SSP) in-water sports athletes (WSA, i.e. those competing in sports that are practiced on [e.g. rowing] or in [e.g. swimming; water polo] water). Indeed, incoherent findings have been observed across individual studies making it difficult to provide the scientific community and coaches with consistent evidence. As such, a comprehensive systematic literature search should be conducted to clarify the existent evidence, identify the major gaps in the literature, and offer recommendations for future studies.

**Aim:**

To examine the effects of PJT compared with active/specific-active controls on the PF (one-repetition maximum back squat strength, squat jump height, countermovement jump height, horizontal jump distance, body mass, fat mass, thigh girth) and SSP (in-water vertical jump, in-water agility, time trial) outcomes in WSA, through a systematic review with meta-analysis of randomized and non-randomized controlled studies.

**Methods:**

The electronic databases PubMed, Scopus, and Web of Science were searched up to January 2022. According to the PICOS approach, the eligibility criteria were: (population) healthy WSA; (intervention) PJT interventions involving unilateral and/or bilateral jumps, and a minimal duration of ≥ 3 weeks; (comparator) active (i.e. standard sports training) or specific-active (i.e. alternative training intervention) control group(s); (outcome) at least one measure of PF (e.g. jump height) and/or SSP (e.g. time trial) before and after training; and (study design) multi-groups randomized and non-randomized controlled trials. The Physiotherapy Evidence Database (PEDro) scale was used to assess the methodological quality of the included studies. The DerSimonian and Laird random-effects model was used to compute the meta-analyses, reporting effect sizes (ES, i.e. Hedges’ *g*) with 95% confidence intervals (95% CIs). Statistical significance was set at *p* ≤ 0.05. Certainty or confidence in the body of evidence for each outcome was assessed using Grading of Recommendations Assessment, Development, and Evaluation (GRADE), considering its five dimensions: risk of bias in studies, indirectness, inconsistency, imprecision, and risk of publication bias.

**Results:**

A total of 11,028 studies were identified with 26 considered eligible for inclusion. The median PEDro score across the included studies was 5.5 (moderate-to-high methodological quality). The included studies involved a total of 618 WSA of both sexes (330 participants in the intervention groups [31 groups] and 288 participants in the control groups [26 groups]), aged between 10 and 26 years, and from different sports disciplines such as swimming, triathlon, rowing, artistic swimming, and water polo. The duration of the training programmes in the intervention and control groups ranged from 4 to 36 weeks. The results of the meta-analysis indicated no effects of PJT compared to control conditions (including specific-active controls) for in-water vertical jump or agility (ES =  − 0.15 to 0.03; *p* = 0.477 to 0.899), or for body mass, fat mass, and thigh girth (ES = 0.06 to 0.15; *p* = 0.452 to 0.841). In terms of measures of PF, moderate-to-large effects were noted in favour of the PJT groups compared to the control groups (including specific-active control groups) for one-repetition maximum back squat strength, horizontal jump distance, squat jump height, and countermovement jump height (ES = 0.67 to 1.47; *p* = 0.041 to < 0.001), in addition to a small effect noted in favour of the PJT for SSP time-trial speed (ES = 0.42; *p* = 0.005). Certainty of evidence across the included studies varied from very low-to-moderate.

**Conclusions:**

PJT is more effective to improve measures of PF and SSP in WSA compared to control conditions involving traditional sport-specific training as well as alternative training interventions (e.g. resistance training). It is worth noting that the present findings are derived from 26 studies of moderate-to-high methodological quality, low-to-moderate impact of heterogeneity, and very low-to-moderate certainty of evidence based on GRADE.

*Trial registration* The protocol for this systematic review with meta-analysis was published in the Open Science platform (OSF) on January 23, 2022, under the registration doi 10.17605/OSF.IO/NWHS3 (internet archive link: https://archive.org/details/osf-registrations-nwhs3-v1).

**Supplementary Information:**

The online version contains supplementary material available at 10.1186/s40798-022-00502-2.

**Key Points**

- Plyometric jump training is an effective method to improve measures of physical fitness (i.e. muscle strength and muscle power) and sport-specific performance (e.g. sport-specific time-trial speed) in-water sports athletes.
- The results of this study are based on a total of 618 water sport athletes, from 26 articles of moderate-to-high methodological quality, low-to-moderate impact of heterogeneity, and very low-to-moderate certainty of evidence.

## Introduction

All sports that are practiced on (e.g. rowing) or in (e.g. swimming; triathlon; water polo; synchronized swimming) water are considered water sports [1, 2]. Many water sports are part of the Olympic games [3, 4]. Water sports are physically demanding [5–9]. Therefore, to successfully cope with such high demands, water sport athletes (WSA) invest many hours of training per minute of competition [10]. For example, international medal-winning rowers spent ~ 1100–1200 h of training per year [11]; if these rowers practiced continuously for the 52 weeks of the year, this would represent an average of ~ 23 weekly hours of training. Therefore, the allocated time should be devoted towards optimal and time-efficient training activities [12]. Such training activities should consider the development of adequate physical fitness (PF) and sport-specific performance (SSP) components, usually involving highly developed muscular fitness [5–9] and aerobic endurance (e.g. cardiorespiratory fitness) [5]. In fact, muscle strength and power (e.g. jumping from the starting block and flip turns in swimmers) play a relevant role in competitive performance [13–15]. For example, up to large associations (*r* = 0.40 to 0.70) have been reported between 20- and 50-m front crawl swimming performance and mean propulsive power in jump squat [16], leg extension strength [17], and horizontal jump distance [18]. More experienced (older) rowers show greater power than younger rowers, and in junior rowers, greater power is shown in international-ranked rowers than in non-ranked rowers [19]. High lower limb muscle power was associated with effective free throws in-water polo [20]. Cycling peak power and vertical jump are positively associated with performance in sailing [21]. In addition to muscle strength and power, training activities should also consider the development of adequate body composition, as performance in-water sports relates to athlete’s body composition (e.g. faster swimmers and rowers usually have greater lean body mass or thigh perimeter) [8, 22, 23]. Moreover, strength and power-related training can also enhance WSA efficiency and competitive velocity [24].

In this regard, higher-intensity shorter-duration training approaches have been recommended over traditional low-intensity high-volume training methods [25–28]. Plyometric jump training (PJT), a high-intensity short-duration training method, may offer an adequate stimulus to improve PF and SSP [29–36], including (but not limited to) muscle strength [37], muscle power [38], and body composition [30, 32]. Training intensity seems to be a key feature of PJT programming [39, 40], with PJT exercises usually implicating a fast stretch–shortening cycle muscle action, allowing greater concentric work performance than an isolated concentric muscle action, stimulating a high rate of force development, and force absorption muscle capacities (i.e. eccentric force) [29, 41, 42]. In addition, PJT implicates muscle stimulus inducing neuro-mechanical adaptations [29] that may be reproduced in both isoinertial (e.g. land) and isokinetic (e.g. water) environments [43–51]. Indeed, PJT may improve WSA performance [31, 52, 53], targeting key muscles from ankle, knee, and hip joints that may aid during key competitive movements such as kicking in swimming [54], jumping from the start platform and flip turns, lower-limb extension during the stroke in rowing [5, 7], among others. Furthermore, PJT may be equally or even more effective to improve PF (e.g. vertical jump; endurance; bone mass) and SSP (e.g. sport-specific sprinting) in WSA compared to other modes of training-sports [55, 56]. Moreover, PJT may improve flexibility [57–59] which is of paramount importance for athletes in general and WSA in particular [54, 60–64]. Although previous intervention studies have evidenced the effectiveness of PJT on PF and SSP in WSA, most published studies have included relatively small samples (i.e. median *n* = 11), a common issue in the sport-science literature [65], casting doubts on the transferability of such findings into practice. Additionally, not all studies [66] agree with the beneficial effects of PJT on PF and SSP in WSA, reflecting inconsistency in the literature.

Previous works have been performed to solve controversy by systematically aggregating the literature related to strength and conditioning practices in WSA [52, 53, 67, 68]. However, most of these reviews did not consider including a meta-analysis, or addressed a restricted population (e.g. only swimmers; only rowers). Moreover, the aforementioned reviews focused on a myriad of strength and conditioning methods, or on very specific and isolated outcomes (e.g. swimming speed), precluding a more comprehensive view regarding the intervention effects. This leaves the question of the effects of single-mode PJT unanswered. Furthermore, when PJT studies were included, the number was low (*n* = 6 studies [53]; *n* = 4 studies [52]; *n* = 2 studies [68]). To account for the previous limitations (i.e. reduced sample size), a meta-analysis seems to be needed to help practitioners taking evidence-based informed decisions as to PJT implementation [69]. Additionally, a systematic review with meta-analysis may help to detect gaps and limitations in the PJT literature, providing valuable information for scientists and practitioners about future research avenues. However, to the authors’ knowledge, no review has attempted to meta-analyse the large amount of currently available literature regarding the potential effects of PJT on PF and SSP in WSA. Therefore, the primary aim of this systematic review with meta-analysis was to examine the effects of PJT, compared with active (i.e. standard sports training) or specific-active (i.e. alternative training intervention) control groups, on the PF (muscle strength, muscle power, body composition) and SSP (in-water vertical jump, in-water agility, time trial) outcomes in WSA, through a systematic review with meta-analysis of randomized and non-randomized controlled studies.

## Methods

### Procedures

A systematic review with meta-analysis was conducted following the guidelines of the Preferred Reporting Items for Systematic Reviews and Meta-Analyses (PRISMA) [70], and adapted a posteriori to new reporting guidelines (e.g. PRISMA 2020) [71] as such changes are expected as the field evolves (e.g. new databases; new concepts/terms). The most relevant adaptations are described in Additional file 1: Table S1.

### Literature Search: Administration and Update

We considered recommendations from the two most comprehensive scoping reviews that previously examined PJT literature [42, 72]. Computerized literature searches were conducted in the electronic databases PubMed, Web of Science, and SCOPUS. The search strategy was conducted using (in different combinations) the Boolean operators AND/OR with the following keywords (all database fields used): “ballistic”, “complex”, “cycle”, “explosive”, “force”, “plyometric”, “shortening”, “stretch”, “training”, and “velocity”. Examples of combinations included: “ballistic” AND “training”; (“ballistic” OR “plyometric” OR “explosive”) AND “training”. Additionally, using the title database field, the following keywords were employed in the search: “jump”, “power”, and “training”. After an initial search in April 2017, an account was created by one of the authors (RRC) in each of the respective databases, through which the author received automatically generated email updates regarding the search terms used. The search was refined in May 2019 and August 2021, with updates received daily (if available). Studies were eligible for inclusion up to January 2022. The main advantage of this search approach is that it assumes that new knowledge will appear and allow improvements in sport/clinical decision-making. Indeed, the rate of PJT studies increased exponentially during the last years [42, 72], and we plan to update this systematic review every 5 years. The same author (RRC) conducted the initial search and removed duplicates. Thereafter, the search results were analysed according to the eligibility criteria (Table 1). The search strategy (code line) for each database and background of search history is described in Additional file 1: Table S1.

**Table 1:** Selection criteria used in the meta-analysis

Category	Inclusion criteria	Exclusion criteria
Population	Healthy water sport athletes, with no restrictions on their fitness or competitive level, sex, or age	Participants with health problems (e.g. injuries, recent surgery), precluding participation in a plyometric jump training programme
Intervention	A plyometric jump training programme, with a minimal duration of ≥ 3 weeks, which included unilateral and/or bilateral jumps, which commonly utilize a pre-stretch or countermovement stressing the stretch–shortening cycle	Exercise interventions not involving plyometric jump training (e.g. upper-body plyometrics only training interventions) or exercise interventions involving plyometric jump training programmes representing less than 50% of the total training load (i.e. volume, e.g. number of exercises) when delivered in conjunction with other training interventions (e.g. high-load resistance training)
Comparator	Active control group (i.e. athletes participating in regular training schedules) Studies comparing different plyometric jump training approaches (e.g. different intensity) without active control group, or traditional control group (i.e. non-active participants) will also be considered, as well as specific-active control groups (e.g. involving alternative training methods such as high-load resistance training)	Absence of control group
Outcome	At least one measure related to physical fitness (e.g. countermovement jump height; body fat) and/or sport-specific performance (e.g. 50-m swimming speed) before and after the training intervention	Lack of baseline and/or follow-up data
Study design	Multi-arm trials	Single-arm trials/observational studies

In selecting studies for inclusion, a review of all relevant titles was conducted before examination of the abstracts and full-text versions. Two authors (RRC and RKT) independently screened the titles, abstracts, and full-text versions of retrieved studies. During the search and review process, potential discrepancies between the same two authors regarding inclusion and exclusion criteria (e.g. type of control group, intervention adequacy) were resolved through consensus with a third author (APC). From selected articles to be included, reference lists were analysed to identify any additional relevant studies.

### Inclusion and Exclusion Criteria

A PICOS (participants, intervention, comparators, outcomes, and study design) approach was used to rate studies for eligibility [70]. Table 1 indicates our inclusion/exclusion criteria.

Additionally, only full-text, peer-reviewed, original studies were considered for the present meta-analysis. Additional exclusion criteria are provided as Additional file 2: Table S2. Because of the potential difficulties of translating articles written in different languages and the fact that 99.6% of the jump training literature is published in English [72], only articles written in English, as well as Spanish, German, and Portuguese (i.e. the authors’ native languages), were considered for this meta-analysis.

### Data Extraction

The extraction of dependent variables from the included studies considered previous recommendations regarding relevant PF and SSP attributes for WSA [5–9, 73–78]. Therefore, the effects of PJT (compared to controls) on either in/on land/water were identified through PF and SSP attributes. Although an extensive list of outcome data to be collected was considered a priori (e.g. maximum oxygen consumption [VO_2max_]; balance), the final list depended on the available number of studies reporting data for a given outcome. Therefore, measures of PF included maximal strength (i.e. one-repetition maximum [1RM] back squat), horizontal jump distance, squat jump height, countermovement jump height, body mass, fat mass, and thigh girth. Additionally, SSP measures included in-water vertical jump height, in-water agility velocity, and time-trial velocity. Jump, linear sprint, change-of-direction speed, and strength testing usually present very high test–retest reliability (with an intra-class correlation coefficient of > 0.9) [79–82], which is essential to ensure strong consistency between analysed studies within a meta-analysis [70]. Independently of the above, when reported, we extracted reliability measures from the included studies (e.g. intra-class correlation coefficient).

The means and standard deviation of dependent variables were extracted at pre- and post-PJT time points from included studies using Microsoft Excel (Microsoft Corporation, Redmond, WA, USA). When the required data were not clearly or completely reported, authors of the respective study were contacted for clarification. If no response was obtained from the authors (after two attempts) or the authors could not provide the requested data, the study outcome was excluded from the analysis. However, when data were displayed in a figure and no numerical data were provided by authors after being contacted, validated (*r* = 0.99, *p* < 0.001) [83] software (WebPlotDigitizer, version 4.5; https://apps.automeris.io/wpd/) was used to derive numerical data from figures by two independent authors (RKT and JA) and the Cronbach’s Alpha was then calculated. Two authors (RRC and RKT) performed data extraction independently, and any discrepancies between them (e.g. mean value for a given outcome, number of participants in a group) were resolved through consensus with a third author (APC).

Data extracted regarding PJT intervention characteristics included (i) the box height used during PJT exercises, (ii) whether the PJT was combined with another lower-limb training method, (iii) duration (number of weeks) of the PJT intervention; (iv) frequency of PJT sessions (sessions per week); (v) intensity of the PJT exercises; (vi) number of total jumps completed during the PJT intervention; (vii) progressive overload applied during the PJT intervention; (viii) recovery time between sets, repetitions, and training sessions; (ix) replacement of a given part of the standard sport training schedule with PJT exercises; (x) type of PJT exercises; and (xi) type of surface used during PJT. We also extracted data regarding participant’s sex, age (years), body mass (kg), height (m), previous experience with PJT, type and level of water sport practiced, and training period of the season.

### Methodological Quality of the Included Studies

The Physiotherapy Evidence Database (PEDro) scale was used to assess the methodological quality of the included studies, which were rated from 0 (lowest quality) to 10 (highest quality). The validity and reliability of the PEDro scale have been established previously [84–86]. Moreover, the PEDro scale is probably the most frequently used in the PJT literature [42, 87, 88]. Its items mostly assess factors related to the risk of bias in studies. Accordingly, it helps to make comparisons between meta-analyses. Considering that it is not possible to satisfy all scale items in PJT interventions [89], as outlined in previous systematic reviews in the sub-field of PJT, the methodological quality of PJT studies was interpreted using the following convention [35, 87, 90]: ≤ 3 points was considered as “poor” quality, 4–5 points was considered as “moderate” quality, and 6–10 points was considered as “high” quality. If trials were already rated and listed in the PEDro database, the respective scores were adopted. Two authors (RRC and RKT) assessed the methodological quality for each included study independently, and any discrepancies between them were resolved via consensus with a third author (APC).

### Summary Measures, Synthesis of Results, and Publication Bias

Although meta-analyses can be done with as few as two studies [91], because reduced sample sizes are common in the sport-science literature [92], including PJT studies [42, 65, 72, 93], meta-analysis was only conducted in the present case when ≥ 3 studies were available [76, 94]. Effect sizes (ES, i.e. Hedges’ *g*) for each PF and SSP attribute in the PJT and control/comparator groups were calculated using pre-training and post-training mean and standard deviation. Data were standardized using post-intervention standard deviation values. The random-effects model was used to account for differences between studies that might affect the PJT effect [95, 96]. The ES values are presented with 95% confidence intervals (95% CIs). Calculated ES were interpreted using the following scale: < 0.2 trivial, 0.2–0.6 small, > 0.6–1.2 moderate, > 1.2–2.0 large, > 2.0–4.0 very large, > 4.0 extremely large [97]. In studies including more than one intervention group, the sample size in the control group was proportionately divided to facilitate comparisons across multiple groups [98]. The level of heterogeneity was assessed using the *I*^2^ statistic, with values of < 25%, 25–75%, and > 75% representing low, moderate, and high levels of heterogeneity, respectively [99]. The risk of publication bias was explored for continuous variables (≥ 10 studies per outcome) [100–102] using the extended Egger’s test [102]. To adjust for publication bias, a sensitivity analysis was conducted using the trim and fill method [103], with L0 as the default estimator for the number of missing studies [104]. All analyses were carried out using the Comprehensive Meta-Analysis software (version 2, Biostat, Englewood, NJ, USA). Statistical significance was set at *p* ≤ 0.05.

#### Moderator Analyses

Using a random-effects model and independent computed single factor analysis, potential sources of heterogeneity likely to influence the effects of training were selected, including participants' sex, type of sport, programme duration (number of weeks), and total number of training sessions. When appropriate, subgroup analyses and single training factor analyses were divided using the median split technique [105–107]. The median was calculated if at least three studies provided data for a given moderator category. Of note, when two experimental groups (with the same information for a given moderator) were included in a study, only one of the groups was considered to avoid an augmented influence of the study on the median calculation. In addition, instead of using a global median value for a given moderator (e.g. median number of weeks, derived from all included studies), median values were calculated considering only those studies that provided data for the outcome being analysed.

### Additional Analyses

#### Certainty of Evidence

Two authors (JA and RRC) judged the certainty of evidence using the Grading of Recommendations Assessment, Development and Evaluation (GRADE) [108–110]. Evidence started at a high level of certainty (per outcome), but was downgraded based on the following criteria: (i) *Risk of bias in studies*: judgments were downgraded by one level if the average PEDro scores were moderate (< 6) or by two levels if they were poor (< 4); (ii) *Indirectness*: low risk of indirectness was attributed by default due to the specificity of populations, interventions, comparators and outcomes being guaranteed by the eligibility criteria; (iii) *Risk of publication bias*: downgraded by one level if there was suspected publication bias; (iv) *Inconsistency*: judgments were downgraded by one level when the impact of statistical heterogeneity (*I*^2^) was high (> 75%); (v) *Imprecision*: one level of downgrading occurred whenever < 800 participants were available for a comparison [111] and/or if there was no clear direction of the effects. In case both were observed, certainty was downgraded by two levels.

When the number of comparison trials was insufficient to perform meta-analysis, the evidence was automatically judged as very low certainty. Therefore, for the outcomes not included in the meta-analyses, the certainty of evidence should be considered very low.

## Results

### Study Selection

**Fig. 1:**
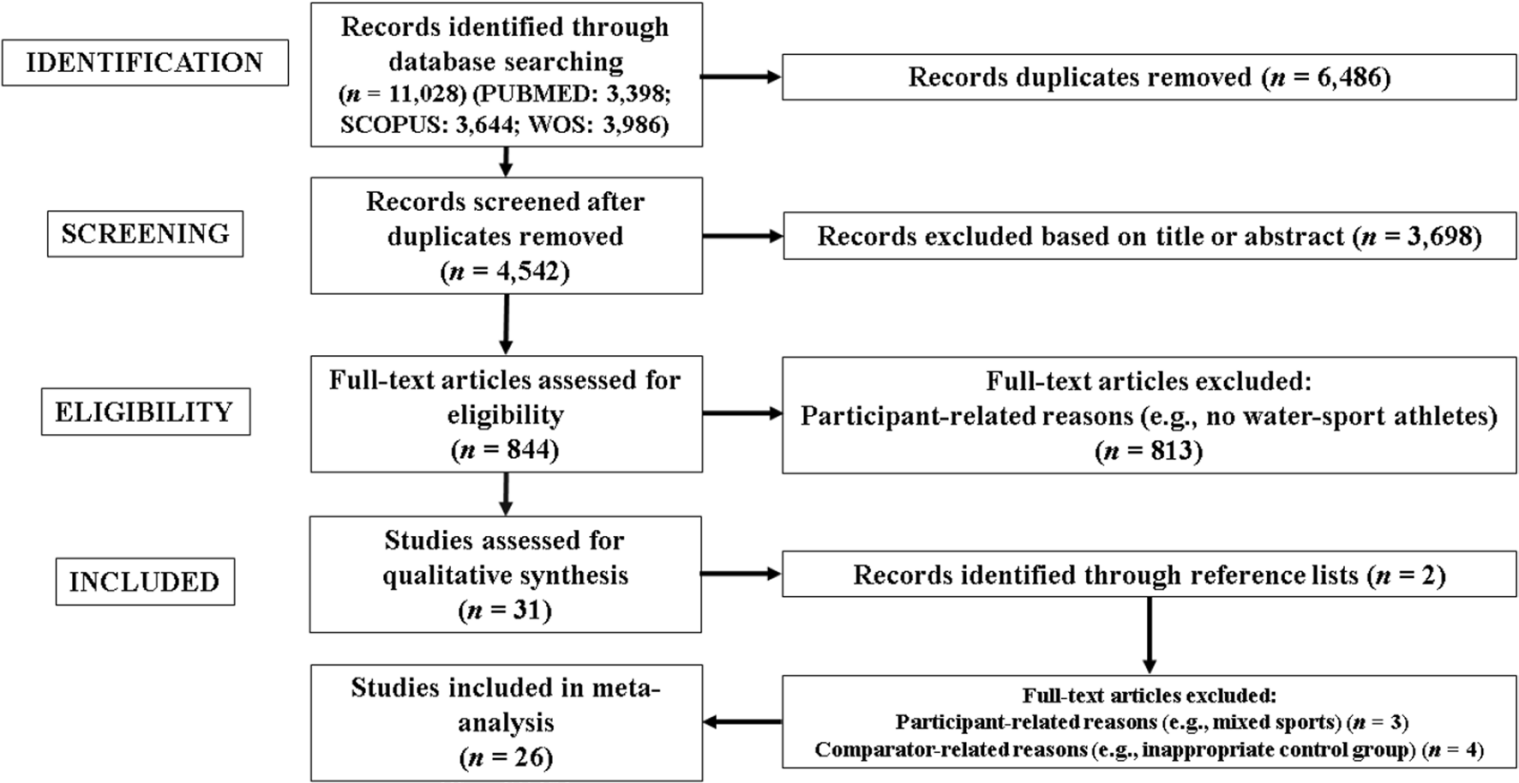
Flow diagram of the search process

The search process in the databases identified 11,028 studies. Figure 1 provides a flowchart illustrating the study selection process.

Duplicate studies were removed (*n* = 6486). After study titles and abstracts were screened, 3,698 studies were removed and 844 full-text studies were screened. Thirty-one studies were included in qualitative synthesis and their reference lists were screened, with 2 records [112, 113] identified through the reference lists screening process. Thereafter, 7 full-text studies were excluded [114–120] (exclusion reasons in Additional file 3: Table S3). Finally, 26 were considered eligible for meta-analysis [56, 66, 112, 113, 121–142].

### Methodological Appraisal of the Included Studies

According to the PEDro checklist, the median (i.e. nonparametric) score was 5.5, with 13 studies attaining high quality (6 points), and 13 studies were of moderate quality (4–5 points). Of note, no study scored above 6 points (Table 2). The two independent reviewers that performed the methodological appraisal of the included studies achieved a Spearman correlation (i.e. nonparametric data) agreement of 0.93.

**Table 2:** Methodological quality of the included studies using the PEDro rating scale

	1	2	3	4	5	6	7	8	9	10	11	Score^a^	Study quality
Amaro et al. [121]	1	1	0	1	0	0	0	1	1	1	1	6	High
Aurell-Badenas et al. [122]	1	0	0	1	0	0	0	1	1	1	1	5	Moderate
Bellver et al. [123]	1	0	0	0	0	0	0	1	1	1	1	4	Moderate
Bishop et al. [124]	1	1	0	0	0	0	0	1	1	1	1	5	Moderate
Bonacci et al. [125]	1	1	0	1	0	0	0	1	1	1	1	6	High
Born et al. [126]	1	1	0	1	0	0	0	1	1	1	1	6	High
Breed and Young [127]	1	1	0	0	0	0	0	1	1	1	1	5	Moderate
Cañas-Jamett et al. [128]	1	1	0	1	0	0	0	1	1	1	1	6	High
Cossor et al. [66]	0	0	0	0	0	0	0	1	1	1	1	4	Moderate
Egan-Shuttler et al. [129]	1	0	0	1	0	0	0	1	1	1	1	5	Moderate
Egan-Shuttler et al. [130]	1	0	0	1	0	0	0	1	1	1	1	5	Moderate
Garrido et al. [131]	1	0	0	1	0	0	0	1	1	1	1	5	Moderate
Jones et al. [132]	1	0	0	0	0	0	0	1	1	1	1	4	Moderate
Kramer et al. [133]	0	1	0	0	0	0	0	1	1	1	1	5	Moderate
Martin et al. [134]	1	1	0	1	0	0	0	1	1	1	1	6	High
Oranchuk et al. [135]	1	1	0	1	0	0	0	1	1	1	1	6	High
Potdevin et al. [136]	1	0	0	1	0	0	0	1	1	1	1	5	Moderate
Pupišová et al. [137]	0	0	0	0	0	0	0	1	1	1	1	4	Moderate
Ramos-Veliz et al. [138]	1	1	0	1	0	0	0	1	1	1	1	6	High
Rebutini et al. [139]	1	0	0	1	0	0	0	1	1	1	1	5	Moderate
Saez de Villarreal et al. [113]	1	1	0	1	0	0	0	1	1	1	1	6	High
Saez de Villarreal et al. [112]	1	1	0	1	0	0	0	1	1	1	1	6	High
Sammoud et al. [141]	1	1	0	1	0	0	0	1	1	1	1	6	High
Sammoud et al. [140]	1	1	0	1	0	0	0	1	1	1	1	6	High
Veliz et al. [142]	1	1	0	1	0	0	0	1	1	1	1	6	High
Vlachopoulos et al. [56]	1	1	0	1	0	0	0	1	1	1	1	6	High

A detailed explanation for each PEDro scale item can be accessed at https://www.pedro.org.au/english/downloads/pedro-scale. In brief: item 1, eligibility criteria were specified; item 2, participants were randomly allocated to groups; item 3, allocation was concealed; item 4, the groups were similar at baseline; item 5, there was blinding of all participants regarding the plyometric jump training programme being applied; item 6, there was blinding of all coaches responsible for the application of plyometric jump training programme regarding its aim towards the improvement of physical fitness/sport-specific performance; item 7, there was blinding of all assessors involved in measurement of physical fitness/sport-specific performance attributes; item 8, measures of at least one key outcome variable were obtained from more than 85% of participants initially allocated to groups; item 9, all participants for whom outcome variables were available received the treatment or control condition as allocated or, data for at least one key outcome variable were analysed by “intention to treat”; item 10, the results of between-group statistical comparisons were reported for at least one key outcome variable; and item 11, point measures and measures of variability for at least one key outcome variable were provided

^a^From a possible maximal score of 10

### Study Characteristics

The participant characteristics and the PJT programmes of the included studies are detailed in Tables 3 and 4, respectively.

**Table 3:** Participants’ characteristics from the included studies

	Sex	Age (y)	BM (kg)	Height (cm)	SPT	Sport	Fit	TP
Amaro et al. [121]	M	12.7	47.7	157.5	No	Swimmers	High	IS
Aurell-Badenas et al. [122]	Mix	26.0	69.1	173.0	No	Triathletes	Normal–high	IS
Bellver et al. [123]	F	19.0	56.3	170.3	No	Artistic swimmers	High	IS
Bishop et al. [124]	NR	13.0	50.6	162.9	NR	Swimmers	Moderate–high	PS
Bonacci et al. [125]	Mix	22.0	65.3	175.5	No	Triathletes	Moderate–high	NR
Born et al. [126]^a^	Mix	17.0	62.9	172.0	No	Swimmers	High	PS
Breed and Young [127]	F	18.9	64.9	166.0	No	Swimmers	Low	NA
Cañas-Jamett et al. [128]	M	20.5	74.8	176.0	No	Swimmers	Normal	NA
Cossor et al. [66]	Mix	11.7	47.4	159.1	NR	Swimmers	Normal–moderate	IS
Egan-Shuttler et al. [129]^a^	M	16.0	71.4	179.0	No	Rowers	Moderate	IS
Egan-Shuttler et al. [130]^a^	M	16.0	71.4	179.0	NR	Rowers	Moderate	IS
Garrido et al. [131]^a^	Mix	12.0	41.3	151.0	No	Swimmers	High	NR
Jones et al. [132]	M	18.9	77.1	178.0	NR	Swimmers	High	PS
Kramer et al. [133]^a^	F	21.3	66.5	170.0	Yes	Rowers	Moderate–high	OS
Martin et al. [134]^a^	M	19.0	83.1	183.0	NR	Water polo	High	IS
Oranchuk et al. [135]^a^	Mix	20.5	69.4	174.0	Yes	Swimmers	Moderate	PS
Potdevin et al. [136]	Mix	14.0	50.0	161.0	NR	Swimmers	Moderate	PS
Pupisova et al. [137]	Mix	17.3	65.8	174.2	No	Swimmers	NR	NR
Ramos-Velis et al. [138]	M	20.4	81.4	180.3	NR	Water polo	High	IS
Rebutini et al. [139]	Mix	22.0	64.9	174.0	NR	Swimmers	High	NR
Saez de Villarreal et al. [113]^a^	M	19.7	80.6	183.3	NR	Water polo	High	PS
Saez de Villarreal et al. [112]^a^	M	23.4	77.6	182.6	NR	Water polo	High	PS
Sammoud et al. [141]	M	10.0	36.2	143.0	NR	Swimmers	Moderate–high	IS
Sammoud et al. [140]	F	10.0	36.4	146.9	NR	Swimmers	Moderate–high	IS
Veliz et al. [142]	F	26.4	72.4	172.3	NR	Water polo	High	IS
Vlachopoulos et al. [56]	M	15.0	57.2	170.3	NR	Swimmers	Normal–high	NR

Abbreviations ordered alphabetically

*BM* body mass, *F* female, *Fit* fitness level (specific to the participants sport) before the experimental intervention, *IS* in-season, *M* male, *n* number of participants per group, *NA* not applicable, *NR* no reported, *OS* off-season, *PS* pre-season, *SPT* systematic experience with plyometric jump training before the experimental intervention, *TP* training period of the season

^a^Denotes that the study included specific-active controls (i.e. alternative training intervention controls), involved in a non-plyometric jump training intervention (e.g. resistance training). The rest of the studies included active controls, involved in their regular sport-specific training schedules

**Table 4:** Plyometric jump training programming variables

	Freq	Dur	Int	BH	NTJ	Tply	Comb	Recov	Tsurf	PO	Repl	Taper
Amaro et al. [121]	2	6	NR	30	504/720 s^a^	BJ	No	40–90/NR/NR	Land	V	No	No
Aurell-Badenas et al. [122]	3	8	NR	NR	3012	Mix	RT	45/NR/ ≥ 48	Land	T/V	NR	No
Bellver et al. [123]	2	22	Jump rate	NA	28,828	Jump rope	VT	0–90/NA/NR	Land	V	NR	No
Bishop et al. [124]	2	8	Height	43–64	1768	Mix	No	60–90/NR/NR	Land	Int/T/V	No	No
Bonacci et al. [125]	2–3	8	Max	NA	1221/1650 m	Mix	RT	NR/NR/NR	Land (mix)	T/V	No	No
Born et al. [126]	2	6	RPE	75–96	1492	BJ	No	150/NR// ≥ 48	Land	Int	Yes	Yes
Breed and Young [127]	3	9	Load	45–60	408	DJ/loaded jump	RT	NR/NR/NR	Land	Int/T/V	No	No
Cañas-Jamett et al. [128]	2	6	Max	20–60	960	Mix	No	60–120/5–10/ ≥ 48	Land (wood)	Int/V	Yes	No
Cossor et al. [66]	3	20	Low/mod	NR	18–27,000	NR	No	NR/NR/48–72	Land	NR	NR	No
Egan-Shuttler et al. [129]	3	4	NR	NR	1705	Mix (vert)	No	NR/NR/48–72	Land	V	Yes	No
Egan-Shuttler et al. [130]	3	4	NR	NR	1705	Mix (vert)	No	NR/NR/48–72	Land	V	Yes	NR
Garrido et al. [131]	2	8	NR	30	350	CMJ/BJ	RT	120/NR/NR	Land	V	No	Yes
Jones et al. [132]	3	6	NR	NR	432–900	BJ/jump squat	No	120–180/NR/ ≥ 48	Land	NR	Yes	NR
Kramer et al. [133]	3	9	Max	30	7131	Mix (vert)	No	NR/NR/NR	Land	V	No	No
Martin et al. [134]	2	18	NR	NA	5042	Mix (vert)	No	180/NR/ ≥ 48	Land	Int/V	NR	No
Oranchuk et al. [135]	2	10	Max	NA	412	Loaded	No	120–180/NR/ ≥ 72	Land	Int/V	Yes	Yes
Potdevin et al. [136]	2	6	NR	40	2146	Mix	No	NR/NR/NR	Land	Int/T/V	No	No
Pupisova et al. [137]	3	8	NR	20–70	NR	Mix	No	NR/NR/48–72	Land	T	No	NR
Ramos-Velis et al. [138]	2	18	Individualized	NA	1044	Load–unloaded	RT	NR/NR/NR	Land	Int/V	No	No
Rebutini et al. [139]	2	9	Max	NR	484	Mix (horizontal)	No	48/60–20/ ≥ 48	Land	Int/V	NR	No
Saez de Villarreal et al. [113]	3	6	Individualized	NA	1620	Load–unloaded	RT	NR/NR/48–72	Land	Int/V	No	No
Saez de Villarreal et al. [112]	3	6	Individualized	NA	1620–2316	Mix	RT/No^a^	NR/NR/48–72	Land/water/mix^a^	Int/V/both^a^	NR	No
Sammoud et al. [141]	2	8	Max	20	1360	AH/CMJ	No	90/NR/72–96	Land (grass)	V	Yes	No
Sammoud et al. [140]	2	8	Max	20	1360	AH/CMJ	No	90/NR/72–96	Land (grass)	V	Yes	No
Veliz et al. [142]	2	16	60–80%	NA	1004	Load–unloaded	RT	NR/NR/ ≥ 48	Land	Int/V	No	No
Vlachopoulos et al. [56]	3–4	36	Load	NA	8880	CMJ	No	6 h/NA/NR	Land	Int/V	NR	Yes

Abbreviations ordered alphabetically

*AH* ankle hops, *BH* box (or similar) height (cm) used during PJT exercises, *BJ* box jump, *CMJ* countermovement jump, *Comb* the PJT was combined with another lower-limb training type, such as RT, *DJ* drop jump, *Dur* duration (weeks) of the PJT intervention, *Freq* frequency of PJT sessions (days per week), *Int* intensity of the PJT exercises. For those using maximal intensity (i.e. denoted as *Max* in the column), the intensity index varied depending on the exercise (e.g. height; distance; RSI; power output; time contact; impact force; load for the loaded jumps; percentage of one repetition maximum), *Mod* moderate intensity, *NA* not applicable, *NR* no reported, *NTJ* number of total jumps completed during the PJT intervention, *PJT* plyometric jump training, *PO* progressive overload applied during the PJT intervention, *Recov* recovery time between sets (in seconds, unless stated otherwise), repetitions (seconds), and training sessions (hours), respectively, *Repl* replacement of a given portion of the regular sport-specific training schedule (i.e. *load*) with the PJT intervention, *RPE* rating of perceived effort, *RSI* reactive strength index, *RT* resistance training. Usually involving squat, split squat, leg press, or similar exercises for the lower limbs, *T* technique-type overload (e.g. the exercises varied across time), *Tply* type of PJT exercises, with “Mix” denoting a combination usually between three or more PJT exercises involving vertical, horizontal, unilateral, bilateral, repeated and/or non-repeated PJT exercises, *Tsurf* type of surface used during PJT, *V* volume-based overload (e.g. from 90 jumps per session at week 1, the overload involved 100 jumps per session at week 2), *Vert* vertical, *VT* vibration training

^*^^a^Depending on the experimental group

The 26 included studies recruited swimmers, triathletes, rowers, artistic swimmers, and water polo athletes, for a total of 618 participants, with 330 participants in the intervention groups (31 groups) and 288 participants in the control groups (26 groups). Among the 26 control groups, 9 groups were specific-active controls (see Table 3; i.e. alternative training intervention controls), involved in a non-PJT intervention (e.g. resistance training), and the other 17 groups were active controls, involved in their regular sport-specific training schedules (see Table 3). Twelve studies included participants with a mean age of < 18 years old (Table 3). Regarding participants' sex, one study did not report the sex of the participants (*n* = 22 [4% of total participants]), nine studies reported a mixed sample of male and female participants (*n* = 202 [33% of total participants]), five studies involved females only (*n* = 114 [18% of total participants]), and 11 studies involved male participants (*n* = 280 [45% of total participants]) (Table 3).

The duration of the training programmes in the intervention and control groups ranged from 4 to 36 weeks (Table 4) and the frequency of weekly training sessions ranged from 2 to 4 (Table 4). Methods for reporting training intensity included maximal effort intensity, such as reactive strength index, vertical jump height, horizontal distance, power output (associated with a given external load), or minimal ground-contact time (Table 4). Training intensity was also reported as ground impact force (i.e. *N*), impact load rate (e.g. body mass/s), jumping rate (e.g. 1.8 jumps/s), rating of perceived exertion (e.g. 11–16, using a 20-point maximal scale), load (i.e. kg) for the loaded jumps, and percentage of one-repetition maximum. Some studies (*n* = 2) reported training intensity only qualitatively (e.g. low-moderate intensity), without further quantification, and some studies (*n* = 3) reported an individualized approach for the programming of PJT intensity. Nine studies did not provide any details regarding intensity (Table 4).

The testing protocols for each of the included PF and SSP outcomes in the meta-analysis are detailed in Table 5.

**Table 5:** Measurement protocols for studies outcomes included in meta-analysis

References	Outcome	Procedure
Amaro et al. [121]	CMJ	Vertical jump height (cm) was obtained with the CMJ, using a contact mat connected to an electronic power time (Ergo-jump, Globus, Italy). The average of three valid attempts was taken to analysis, with a 2-min rest between maximal attempts. The ICC was > 0.95
	Time trial	Participants completed two maximal 50-m front crawl attempts (with 15 min of rest between) to access their best time (s). The ICC values ranged from 0.93 to 0.98. The starts were performed in the starting block. Two experienced researchers measured time with a chronometer
Aurell-Badenas et al. [122]	CMJ	Measured (cm) using a contact platform (Optojump Next; Microgate, Bolzano, Italy). Participants were familiarized with the test. The participants were instructed to jump as high as possible whilst maintaining their hands-on hips
	SJ	As above, participants were asked to perform a maximal effort vertical jump from a squat position with the knee flexed at approximately 90° (i.e. without a CMJ)
Bellver et al. [123]	Fat mass	The fat mass (gm) was assessed using dual-energy X-ray absorptiometry (Lunar DXA TM GE Medical Systems, version 12.30). Participants were measured in light clothing, barefoot, and without any jewellery or metal buttons. All subjects went to the toilet before the test. The same technician performs all measurements. Athletes were evaluated in a supine position, with their feet in slight internal rotation to have good visibility of the femoral neck
	Body mass	The body mass was measured in kg
Bishop et al. [124]	Time trial	Each subject’s video footage was uploaded to Silicon Coach Pro (siliconCOACH, Ltd, Dunedin, New Zealand) and subsequently analysed to determine the time (s) to complete a distance of 5.5 m from starting stimulus. The distance was defined with visual reference points on the lane markers and poolside
Bonacci et al. [125]	Body mass	Body mass was measured to the nearest 0.01 kg. The measurements were per the International Society for the Advancement of Kinanthropometry protocols and conducted by a certified level 2 anthropometrist
	Thigh girth	Girth (cm) was measured from the right thigh. The measurements were per the International Society for the Advancement of Kinanthropometry protocols and conducted by a certified level 2 anthropometrist
Born et al. [126]	Time trial	A 25-m swim sprint was performed from the starting block, and the underwater phase was allowed for a maximum of 15 m. All athletes used the kick start technique with inclined rearfoot support. After 2 familiarization trials, the best of 3 trials was used for analysis. Tests were performed in a group of 5 athletes, allowing 4 to 5 min of rest between trials. The 25-m sprint time (s) was measured from the starting signal (light trigger of the starting device visible in the video footage) until the head of the swimmer passed the 25-m mark
Breed and Young [127]	CMJ	For the CMJ, a 78 × 52 cm contact mat linked to a computer to calculate the jump height (cm). Hands were placed on the hips and the participants were instructed to maintain the same body position when landing as during the take-off (i.e. hip, knees, and ankles in an extended position)
Cañas-Jamett et al. [128]	Time trial	Swimmers completed a warm-up of 50 m using the crawl swim style in a 25-m pool, and after 5 min of rest, they performed one maximal 200 m time trial (s). They began the test by jumping from an official platform at the edge of the swimming pool. A digital watch was used to measure the race time
	SJ	A SJ was used to assess maximal vertical jump height (cm) and was performed using an electronic mat system (Ergo-jump, Globus, Italy). During testing, the participants were instructed to place their hands on their hips, with their feet shoulder-width apart, and adopt a flexed ~ 90° knee position for ~ 3 s, followed by a maximal effort vertical jump. Take-off and landing were standardized to full knee and ankle extension on the same spot. Participants were instructed to maximize jump height and bend the knees after landing. 3 trials were completed with a rest period of 2 min. The highest jump was used for the subsequent analysis
	Thigh girth	Thigh girth was assessed 1 cm under the gluteal skinfold and perpendicular to the thigh axis. A non-extensible metallic tape of 0.5 cm width (Lufkin, Executive-Thinline, USA) was used to measure the thigh girth (cm), while participants were standing with their feet shoulder-width apart. Three measurements were carried out for each leg, in a counterbalanced order (i.e. right, left). Since the difference between the first and second measurements was always < 0.5 cm, the mean value between them was used for the analysis
Cossor et al. [66]	Time trial	The subjects completed two hand-timed (s), push-start, maximal effort, 50-m swim
Egan-Shuttler et al. [129]	Time trial	Firstly, rowing economy was measured, following which a 30-min rest was allowed for participants to perform a maximal 500-m time trial (s) on the rowing ergometer (Model D, Concept2, VT, USA). All participants were familiar with performing maximal 500-m trials as these were performed frequently as part of their normal training and/or performance assessments, prior to enrolment in the study, but none were performed during the intervention period
	Thigh girth	The thigh circumference (cm) was measured using Gulick tape. The measurements were taken by the same member of the research team for pre- and post-testing and were taken halfway up the thigh
Egan-Shuttler et al. [130]	Body mass	The participant’s body mass (kg) was measured upon arrival (to the laboratory)
Garrido et al. [131]	Time trial	All the subjects performed two maximal 25-m front crawl trials with a 15-min passive recovery period between the two trials. The evaluation process was conducted in a 25-m indoor swimming pool with in-water starts. The performance time (m/s) was determined by two trained assessors with a chronometer (Golfinho Sports MC 815, Aveiro, Portugal), and the mean value of both measurements was obtained in each trial. The ICC was 0.94
	CMJ	The vertical jump height (cm) was measured using the CMJ. The protocol required the performance of three jumps, each followed by two min of rest. An average of the two best jumps was used for analysis. This test was measured on a trigonometric carpet (Ergo-jump Digitime 1000, Digest Finland). The ICC was 0.92
Jones et al. [132]	Time trial	Each subject performed three maximal effort turns, with a 3-min rest period between each turn. The swimmer swam from 20 m out towards the wall at full speed, undertook their preferred stroke turn, touch, or tumble, and swam at maximal effort back out to the 20-m mark. The time (s) to 5 m post-turn was recorded
Kramer et al. [133]	Time trial	A 2500-m time test (s) was conducted using a Concept IT-Plus Rowing Ergometer. The rowers selected their own stroke rates. However, they all rowed with the chain on the inner sprocket and the air vents closed
Martin et al. [134]	In-water jump	In-water jump was assessed using a board with a cm scale attached to it and a video camera (50-Hz sampling frequency) placed 3 m away from the board. From the floating position, the players were required to jump as high as possible. The subsequent video analysis was performed by freezing the image at the highest point of hand contact on the board. Three trials were completed with 30 s rest between each trial. The mean of the 3 trials was used for further analyses
	In-water agility	Assessed using the 10-m T-agility test. Subjects were instructed to sprint from a standing starting position (upright position facing the far end of the pool) at the base of the T. The test was initiated when the examiner gave the “start” signal, and the athlete’s head crossed the photocell to initiate the timing gate (MuscleLab, version 7.18). The subjects swim 5 m to the goal and touch the crossbar with both hands, then side swim to the right post and touch it, and then side swim to the left post and touch it. After that, they swam 5 m backward until they crossed the photocell. Three trials were completed, with 5 min of rest between trials. The mean of each agility trial time (s) was used for the subsequent statistical analyses
	Time trial	Maximal sprint swim times (s) were recorded for a 20-m distance in a 25-m indoor swimming pool. Subjects were positioned 1 m off the wall (upright position facing the far end of the pool), before they were signalled to start the sprint with a random sound. Infrared timing systems (MuscleLab [version 7.18]) were stationed at the sprint start and endpoints (0 and 20 m). Three trials were completed, with 5 min of rest between trials. The mean of the times achieved across the 3 trials was used for subsequent statistical analyses
	CMJ	The CMJ height (cm) was calculated using an infrared timing system MuscleLab (Ergo-Jump, version 718; Ergotest Technology, Langesund, Norway). Three trials were completed with 2 min rest between each trial. The mean of the 3 trials was then used for subsequent statistical analyses
Orunchuk et al. [135]	CMJ	The athletes performed 5 CMJ with each jump separated by 5 s. The CMJ was performed with a rapid descent to a self-selected depth, immediately followed by a maximal ascent. Athletes were instructed to keep their hands on their hips. All jumps were monitored by the same researcher, and strong verbal encouragement was provided to ensure each jump was performed maximally
	SJ	Athletes performed a knee angle of 90°, measured with a goniometer. This position was held for 3 s before a verbal command to jump was given. An SJ was considered successful if the athlete gave a maximal effort and there was no visible countermovement. Athletes were instructed to keep their hands on their hips. All jumps were monitored by the same researcher, and strong verbal encouragement was provided to ensure each jump was performed maximally
Potdevin et al. [136]	Time trial	Assessed with a 25-m front crawl swim, with a water start without push-off. All the starts were on the initiative of the swimmer. Two independent observers recorded times, and these 2 values were averaged to calculate averaged swimming speed (m/s). The start signals for the water start without push-off the start signal consisted of the swimmer’s limbs moving
	CMJ	The CMJ height (cm) was evaluated using an Ergo-jump (Junghans GMBH-Schramberg, Germany). Three trials were performed, with hands-on hips. Subjects were verbally encouraged to jump with maximal effort. The best performance was retained for statistical analysis
	SJ	As above
	Body mass	Measured with an impedance metric balance scale (Tanita, Tokyo, Japan)
	Fat mass	Estimated with an impedance metric balance scale (Tanita, Tokyo, Japan)
Pupisova et al. [137]	CMJ	Five trials of CMJ were conducted
	SJ	Five trials of SJ were conducted
Ramos-Veliz et al. [138]	Time trial	Maximal sprint swim times were recorded for a 20-m distance, in an indoor swimming pool of 25 m. The participants were positioned 1 m off the wall (upright position facing the far end of the pool) before they were signalled to start the sprint with a random sonorous sound. Infrared beams were stationed at the sprint start and endpoints (0 and 20 m) with time measured to the nearest 0.01 s using an electronic timing system (Muscle Lab.V7.18, Ergotest Technology, Langesund, Norway). Three trials were completed, with 5 min of rest between trials. The shortest time was used for analysis
	1RM squat	The participants performed the full squat from a fully extended position starting with shoulders in contact with the bar. On command, the participants performed a controlled eccentric squat to a knee angle of 60°, followed without pause by a concentric leg extension (as fast as possible) returning to full extension. The trunk was kept as straight as possible and an accredited coach conducted this test and checked for correct technique. A safety belt was used by all the participants. The tests were performed in a squatting apparatus (Smith machine, Model Adan-Sport, Granada, Spain). Five to six separate single attempts were performed until the subject was unable to extend the legs to the required position. The last acceptable lift with the highest possible load was determined as 1 RM. The rest period between trials was 2 min
	CMJ	Assessed with an infrared curtain system (Ergo-Jump, MuscleLabV718, Langesund, Porsgrunn, Norway) to measure flight and contact times. Five trials were completed with 1 min of rest between trials. The 2 extreme values of the 5 trials were eliminated (best and worst), and the mean of the 3 central values was used for the subsequent statistical analysis
Rebutini et al. [139]	SLJ	The horizontal jump displacement was calculated during a swimming block start performance test. Kinematic data were collected using a bi-dimensional approach. A digital video camera (Casio, model EX-FH20, Japan) operating at 210 Hz was perpendicularly positioned approximately 5 m away from the left sagittal plane of the participants. A light-emitting diode (LED) signal allowed to synchronize the kinematic and kinetic data using the instant of take-off as a reference. The markers were manually digitized using commercial software (SIMI Motion Software, version 6.1, Germany), and the coordinates were filtered using as order recursive Butterworth filter with a cut-off frequency set at 8 Hz. Thereafter, the horizontal displacement (cm) of centre of mass from the last block contact to water entrance was determined
Saez de Villareal et al. [113]	In-water jump	The authors cited a previous study to refer to the jump assessment protocol. The ICC was 0.92 (0.90–0.94)
	In-water agility	Participants’ in-water agility was evaluated by using the 10-m T Swimming Agility test using a photocell timing system (Muscle Lab.V7.18). For this test, the athletes were instructed to sprint from a standing start position (from an upright floating position facing the far end of the pool) at the base of the T. Following a starting signal from the investigators, the athlete swam to the goal, touched the crossbar with both hands, then side swam to the right post, touches it before side-swimming to touch the left post. The athlete then swam 5 m backward through photocells. The test score was recorded as the best time (s) of 3 trials. A 3-min rest period was allowed between each trial. The ICC was 0.86 (0.84–0.88)
	Time trial	Maximal 20-m sprint swim times were recorded to the nearest 0.01 s using an electronic timing system (Muscle Lab.V7.18), in an indoor swimming pool of 25 m. Participants were positioned 1 m off the wall (from an upright floating position facing the far end of the pool) before they were signalled to start the sprint with a random start signal. Three trials were completed, with 5 min of rest between trials. The shortest time was used for analysis. The ICC was 0.91 (0.90–0.93)
	CMJ	The countermovement jump (CMJ) test was performed using an infrared curtain system (Ergo-Jump; Muscle Lab.V7.18, Langesund, Norway). Five trials were completed with 1 min of rest allocated between each trial. The 2 extreme values of the 5 trials were eliminated (best and worst), and the mean of the 3 central values was used for the subsequent statistical analysis. The ICC was 0.93 (0.91–0.95)
	1RM squat	Participants performed the full squat from a fully extended position starting with shoulders in contact with the bar. On command, the participants performed a controlled eccentric squat to an internal knee angle of 60°, followed without pause by a concentric leg extension (as fast as possible) returning to full extension. The trunk was kept as straight as possible and an accredited coach conducted this test and checked for correct technique. A safety belt was used by all participants. The tests were performed in a squatting apparatus (Smith machine; Model Adan-Sport, Granada, Spain). Five to six separate single attempts were performed until the subject was unable to extend the legs to the required position. The last acceptable lift with highest possible load was determined as 1 RM. The rest period between trials was 2 min
Saez de Villarreal et al. [112]	In-water jump	The authors cited a previous study to refer to the jump assessment protocol
	In-water agility	Assessed using the 10-m T-agility test. The subjects were instructed to sprint from a standing starting position (upright position facing the far end of the pool) at the base of the T. The test was initiated when the examiner gave the signal to initiate the test and the athlete’s head crossed the photocell to initiate the timing apparatus (Muscle Lab.V7.18, Langesund, Norway). In this test, the subjects were instructed to swim to the goal and touch the crossbar with 2 hands, then side swim to the right post and touch it and then side swim to the left post and touch it. After that, the subject was required to swim 5-m backward until they crossed the photocell and timing was ceased. A 2-min rest period was allowed between each trial. The mean of each agility trial time (s) was used for the subsequent statistical analyses
	Time trial	Maximal sprint swim times were recorded for a 20 m distance in a 25-m indoor swimming pool. Subjects were positioned 1 m off the wall (upright position facing the far end of the pool) before they were signalled to start the sprint with a random sonorous sound. Infrared beams were stationed at the sprint start and endpoints (0 and 20 m) with time measured to the nearest 0.01 s using an electronic timing system (Muscle Lab. V7.18). Three trials were completed, with 2 min of rest between trials. The mean of the times achieved across the 3 trials was used for subsequent statistical analyses
	1RM squat	Participants performed the full squat from an extended position with the bar held across the shoulders with a standardized front squat grip. On command, the subjects performed a controlled eccentric squat to a depth that allowed for the attainment of a 60° (using a goniometer) knee angle. Once this knee angle was achieved, a squat depth that allowed for this knee angle the subjects performed a concentric knee extension motion as fast as possible to return to a fully extended position. All subjects wore a standard lifting belt during each trial. The tests were performed in a Smith machine (Model AdanSport, Granada, Spain). Four to six separate single attempts were performed until the subject was unable to perform each lift with appropriate technique or unable to complete a repetition with the tested load. The last acceptable lift with used to quantify 1 RM. The rest period between trials was 2 min
	CMJ	Assessed with an infrared curtain system (MuscleLab.V718; Ergo-Jump, Langesund, Norway). Three trials were completed with 2 min of rest between each trial. The mean of the 3 trials was then used for subsequent statistical analyses
Sammoud et al. [141]	Time trial	Swimmers performed the 50-m front crawl swimming trials with a diving start. All starts were voluntarily initiated by the swimmers. Two independent observers recorded performance times using stop-watches. The average of the two recorded values was used. The start signal for the observer was the moment as the swimmer’s feet left the block. The distance was standardized using markers at the bottom of the pool. The final signal for the observer was the moment when the swimmer’s hand touched the wall. The ICC ranged between 0.89 and 0.91 and the TEM ranged between 1.2 and 2.5%
	CMJ	CMJ techniques were visually controlled by the first author of this study. Jump height was recorded using an Optojump photoelectric system (Microgate, SRL, Bolzano, Italy). The ICC was 0.98 and the TEM was 2.9%
	SLJ	Participants executed the SLJ with their legs and arms for maximal horizontal distance. Participants had to land with both feet simultaneously, avoiding falling forward or backward. Distance was measured to the nearest cm, between the starting line and the heel of the rear foot, recorded via tape measure. ICC = 0.96; TEM = was 0.5%
	Body mass	The body mass was recorded by a trained anthropometrist assisted by a recorder. Standardized procedures were applied per the International Society for the Advancement of Kinanthropometry
Sammoud et al. [140]	Time trial	Swimmers performed 50-m front crawl trials with a diving start. All starts were voluntarily initiated by the swimmers. Two independent observers recorded performance times using stop-watches. During the diving start tests, participants were not allowed to drift forward or backward before initiating the start. The average of the two recorded values was used for statistical analyses. The start signal for the observer was the moment as the swimmer’s feet left the block. The distance was standardized using markers at the bottom of the pool. The final signal for the observer was the moment when the swimmer’s hands touched the wall. The ICC ranged between 0.89 and 0.91
	CMJ	CMJ techniques were visually controlled by the first author of this study. Jump height was recorded using an Optojump photoelectric system (Microgate, SRL, Bolzano, Italy). The ICC was 0.98
	SLJ	The starting position of the SLJ required subjects to stand with their feet behind a starting line. Participants executed a countermovement with their legs and arms and jumped at maximal effort in horizontal direction. Participants had to land with both feet simultaneously and were not allowed to fall forward or backward. The horizontal distance (cm) between the starting line and the heel of the rear foot was recorded via tape measure to the nearest 1 cm. The ICC for was 0.96
	Body mass	The body mass was assessed by a trained anthropometrist who was assisted by a co-worker. Standardized procedures were applied which were per the International Society for the Advancement of Kinanthropometry
Veliz et al. [142]	In-water jump	The in-water jump was assessed using a board with a cm scale attached to it and a video camera (50-Hz sampling frequency) placed 3 m away from the board. From the floating position the players were required to jump the highest that they could reach. The subsequent video analysis was performed by freezing the image at the highest point of hand contact on the board by the players. Three trials were completed with 2 min of rest between trials. The mean of the 3 values was used for the subsequent statistical analyses
	Time trial	The time trial was recorded for 20-m maximal sprint swim, in an indoor swimming pool of 25 m. The participants were positioned 1 m off the wall (upright position facing the far end of the pool), before they were signalled to start the sprint with a random sonorous sound. Infrared beams were stationed at the sprint start and endpoints (0 and 20 m) with time measured to the nearest 0.01 s using an electronic timing system (Muscle LabV718). The head of the athletes triggered the infrared timing beams. Three trials were completed, with 5 min of rest between trials, and the shortest time was used for the subsequent statistical analysis
	1RM squat	The participants performed the full squat from a fully extended position starting with shoulders in contact with the bar. On command, the participants performed a controlled eccentric squat to a knee angle of 60°, followed without pause by a concentric leg extension (as fast as possible) returning to full extension. The trunk was kept as straight as possible and an accredited coach conducted this test and checked for correct technique. All the participants used a safety belt. The tests were performed in a squatting apparatus (Smith machine, Model Adan-Sport, Granada, Spain). Four to six separate single attempts were performed until the subject was unable to perform each lift with appropriate technique or unable to complete a repetition with the tested load. The last acceptable lift with the highest possible load was determined as 1 RM. The rest period between trials was 2 min
	CMJ	The CMJ test was performed using an infrared curtain system (Ergo-Jump, MuscleLabV718, Langesund, Porsgrunn, Norway). Three trials were completed with 2 min of rest between trials. The mean of the 3 values was used for the subsequent statistical analyses
Vlachopoulos et al. [56]	CMJ	Assessed on a jump mat (Probotics Inc., AL, USA). Three maximal jumps were performed, using the best score
	SLJ	For the SLJ, participants were advised to jump as far as possible to land with both feet and the distance (cm) measured between the starting line and the participant’s heels was recorded. For SLJ, three maximal jumps were performed and the best score was used
	Fat mass	A Lunar Prodigy DXA scanner (GE Healthcare Inc., WI, USA) was used to measure the fat mass (g). All scans were undertaken by the same fully trained operator. The DXA percentage coefficient of variation has been reported between 1.0 and 2.9%

Abbreviations ordered alphabetically

When reliability (e.g. ICC) was reported, the information was included

*CMJ* countermovement jump, *ICC* intra-class correlation coefficient, *SJ* Squat Jump, *SLJ* standing long jump (involves horizontal displacement of the centre of mass), *TEM* typical error of measurement, *1RM* one repetition maximum

Some studies involved results from a single trial [129, 130] (i.e. the studies used the same population). However, as the different publications reported different outcomes, both studies were included in the meta-analysis. In studies including more than one intervention group, the sample size in the control group was proportionately divided to facilitate comparisons across multiple groups [98]. This was also the case for studies involving four experimental groups [134] and two experimental groups [112, 121].

### Results of the Meta-analysis

#### Sport-Specific Performance

**Fig. 2:**
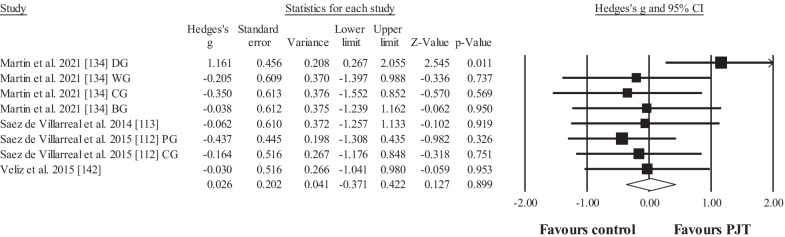
Forest plot for changes regarding in-water vertical jump performance (e.g. cm) in participants after plyometric jump training (PJT) compared to controls. Forest plot values are shown effect sizes (Hedges’ *g*) with 95% confidence intervals (CI). Black squares: individual studies. Its size represents their relative weights. White rhomboid: summary value. *Note*: Letters (e.g. DG) at the end of a study (e.g. Martin et al. [134] DG) denotes that different experimental groups were included

Four studies provided data for in-water vertical jump performance height, involving 8 experimental and 4 control groups (pooled *n* = 126; specific-active control groups, *n* = 3). The results showed no effect for the PJT groups compared to the control groups (ES = 0.03; 95% CI = − 0.37 to 0.42; *p* = 0.899; Fig. 2; *I*^2^ = 12.2%).

**Fig. 3:**
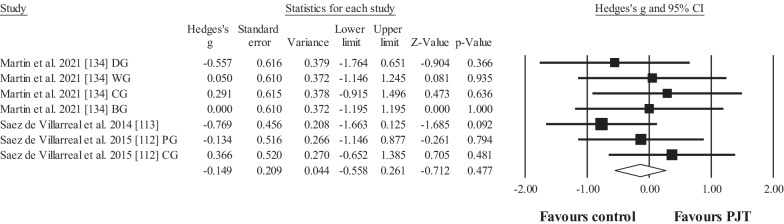
Forest plot for changes regarding in-water agility (e.g. agility time) in participants after plyometric jump training (PJT) compared to controls. Forest plot values are shown effect sizes (Hedges’ *g*) with 95% confidence intervals (CI). Black squares: individual studies. Its size represents their relative weights. White rhomboid: summary value. *Note*: Letters (e.g. DG) at the end of a study (e.g. Martin et al. [134] DG) denotes that different experimental groups were included

Three studies provided data for in-water agility performance (e.g. speed), involving 7 experimental and 3 control groups (pooled *n* = 105; specific-active control groups, *n* = 3). The results showed no effect for the PJT groups compared to the control groups (ES = − 0.15; 95% CI = − 0.56 to 0.26; *p* = 0.477; Fig. 3; *I*^2^ = 0.0%).

**Fig. 4:**
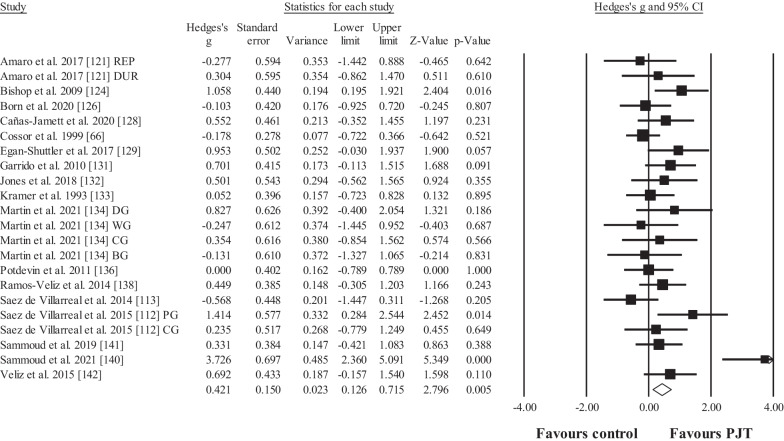
Forest plot for changes regarding in-water time trial performance (e.g. 25-m swimming speed) in participants after plyometric jump training (PJT) compared to controls. Forest plot values are shown effect sizes (Hedges’ *g*) with 95% confidence intervals (CI). Black squares: individual studies. Its size represents their relative weights. White rhomboid: summary value. *Note*: Letters (e.g. DG) at the end of a study (e.g. Martin et al. [134] DG) denotes that different experimental groups were included

Seventeen studies provided data for time trial performance (e.g. speed), involving 22 experimental and 17 control groups (pooled *n* = 438; specific-active control groups, *n* = 8). The results showed a significant small effect in favour of the PJT groups compared to the control groups (ES = 0.42; 95% CI = 0.13 to 0.72; *p* = 0.005; Fig. 4; *I*^2^ = 54.6%; Egger test = 0.051).

#### Physical Fitness

**Fig. 5:**
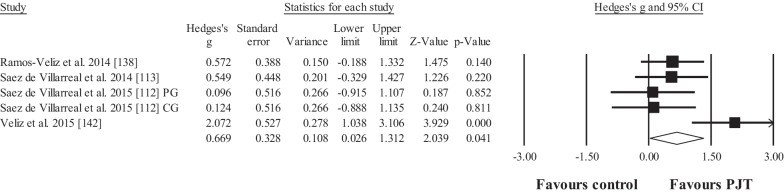
Forest plot for changes in maximal strength performance (i.e. squat one repetition maximum, as kg), in participants after plyometric jump training (PJT) compared to controls. Forest plot values are shown effect sizes (Hedges’ *g*) with 95% confidence intervals (CI). Black squares: individual studies. Its size represents their relative weights. White rhomboid: summary value. *Note*: Letters (e.g. DG) at the end of a study (e.g. Martin et al. [134] DG) denotes that different experimental groups were included

Four studies provided data for maximal strength performance (i.e. one-repetition maximum [1RM] back squat), involving 5 experimental and 4 control groups (pooled *n* = 97; specific-active control groups, *n* = 3). The results showed significant, moderate effect in favour of the PJT groups compared to the control groups (ES = 0.67; 95% CI = 0.03 to 1.31; *p* = 0.041; Fig. 5; *I*^2^ = 58.1%).

**Fig. 6:**
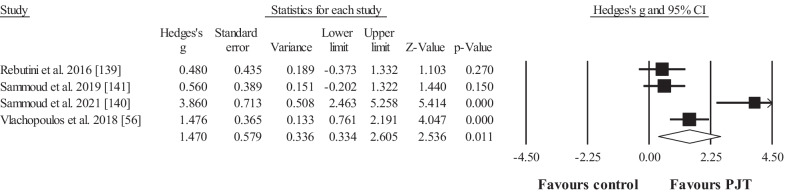
Forest plot for changes in horizontal jump displacement performance (e.g. cm), in participants after plyometric jump training (PJT) compared to controls. Forest plot values are shown effect sizes (Hedges’ *g*) with 95% confidence intervals (CI). Black squares: individual studies. Its size represents their relative weights. White rhomboid: summary value

Four studies provided data for horizontal jump displacement performance, involving 4 experimental and 4 control groups (pooled *n* = 105; specific-active control groups, *n* = 0). The results showed significant, large effect for the PJT groups compared to the control groups (ES = 1.47; 95% CI = 0.33 to 2.61; *p* = 0.011; Fig. 6; *I*^2^ = 84.9%).

**Fig. 7:**
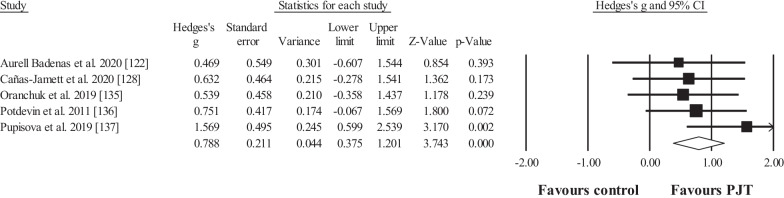
Forest plot for changes in squat jump performance (e.g. vertical height), in participants after plyometric jump training (PJT) compared to controls. Forest plot values are shown effect sizes (Hedges’ *g*) with 95% confidence intervals (CI). Black squares: individual studies. Its size represents their relative weights. White rhomboid: summary value

Five studies provided data for squat jump performance height, involving 5 experimental and 5 control groups (pooled *n* = 91; specific-active control groups, *n* = 1). The results showed significant, moderate effect in favour of the PJT groups compared to the control groups (ES = 0.79; 95% CI = 0.38 to 1.20; *p* < 0.001; Fig. 7; *I*^2^ = 0.0%).

**Fig. 8:**
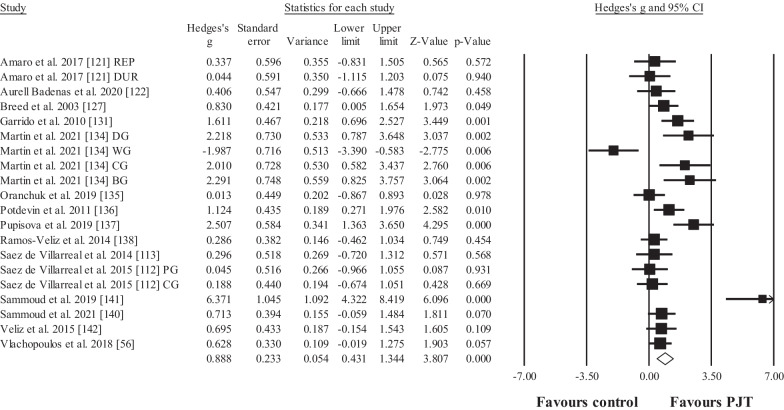
Forest plot for changes in countermovement jump performance (e.g. vertical height), in participants after plyometric jump training (PJT) compared to controls. Forest plot values are shown effect sizes (Hedges’ *g*) with 95% confidence intervals (CI). Black squares: individual studies. Its size represents their relative weights. White rhomboid: summary value. *Note*: Letters (e.g. DG) at the end of a study (e.g. Martin et al. [134] DG) denotes that different experimental groups were included

Fifteen studies provided data for countermovement jump height, involving 20 experimental and 15 control groups (pooled *n* = 378; specific-active control groups, *n* = 8). The results showed significant, moderate effect in favour of the PJT groups compared to the control groups (ES = 0.89; 95% CI = 0.43 to 1.34; *p* < 0.001; Fig. 8; *I*^2^ = 75.9%; Egger test = 0.066).

**Fig. 9:**
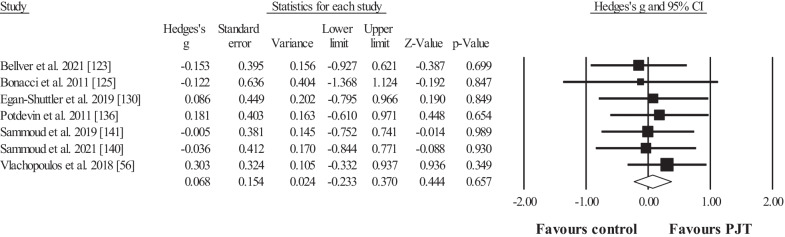
Forest plot for changes in body mass (i.e. kg), in participants after plyometric jump training (PJT) compared to controls. Forest plot values are shown effect sizes (Hedges’ *g*) with 95% confidence intervals (CI). Black squares: individual studies. Its size represents their relative weights. White rhomboid: summary value

Seven studies provided data for body mass, involving 7 experimental and 7 control groups (pooled *n* = 158; specific-active control groups, *n* = 1). The results showed no effect for the PJT groups compared to the control groups (ES = 0.07; 95% CI = − 0.23 to 0.37; *p* = 0.657; Fig. 9; *I*^*2*^ = 0.0%). Similar results (figures not showed) were observed for fat mass and thigh girth (ES = 0.06 to 0.15; *p* = 0.452 to 0.841).

### Moderator Analyses

Participants’ sex, type of sport, programme duration (number of weeks), and total number of training sessions were included as moderators. However, such moderators were available only for the analysis of time-trial performance and countermovement jump height, as the number of studies available for analysis of other outcomes was insufficient.

Regarding time-trial performance changes after PJT, no significant difference was noted between males (14 groups; ES = 0.53, 95% CI = 0.06 to 1.00; *I*^2^ = 63.3%) and females (3 groups; ES = 0.34, 95% CI = − 0.12 to 0.79; *I*^2^ = 0.0%; between-moderators categories *p* value = 0.560). Similarly, no significant difference was noted after < 8 weeks of PJT (10 groups; ES = 0.26, 95% CI =  − 0.10 to 0.61; *I*^2^ = 24.3%) compared to ≥ 8 weeks of PJT (12 groups; ES = 0.56, 95% CI = 0.10 to 1.01; *I*^2^ = 67.1%; between-moderators categories *p* value = 0.303). Further, no significant difference was noted between swimmers (12 groups; ES = 0.46, 95% CI = 0.02 to 0.91; *I*^2^ = 67.9%) and water polo athletes (9 groups; ES = 0.32, 95% CI = -0.06 to 0.71; *I*^2^ = 23.6%; between-moderators categories *p* value = 0.647). Furthermore, no significant difference was noted after < 18 PJT sessions (10 groups; ES = 0.65, 95% CI = 0.11 to 1.18; *I*^2^ = 69.6%) compared to ≥ 18 PJT sessions (12 groups; ES = 0.21, 95% CI =  − 0.08 to 0.51; *I*^2^ = 17.1%; between-moderators categories *p* value = 0.161).

Regarding CMJ height changes after PJT, no significant difference was noted between males (12 groups; ES = 0.53, 95% CI = 0.04 to 1.02; *I*^2^ = 63.9%) and females (3 groups; ES = 2.38, 95% CI = 0.09 to 4.66; *I*^2^ = 92.5%; between-moderators categories p value = 0.123). Further, no significant difference was noted between swimmers (10 groups; ES = 1.19, 95% CI = 0.51 to 1.86; *I*^2^ = 80.2%) compared to water polo athletes (9 groups; ES = 0.61, 95% CI =  − 0.08 to 1.30; *I*^2^ = 73.0%; between-moderators categories *p* value = 0.240). Furthermore, no significant difference was noticed after < 22 PJT sessions (10 groups; ES = 0.85, 95% CI = 0.17 to 1.53; *I*^2^ = 78.4%) and ≥ 22 PJT sessions (10 groups; ES = 0.94, 95% CI = 0.29 to 1.59; I^2^ = 75.6%; between-moderators categories *p* value = 0.849). However, a significant difference was reported after < 8 weeks of PJT (6 groups; ES = 0.39, 95% CI = − 0.01 to 0.80; *I*^2^ = 0.0%) compared to ≥ 8 weeks of PJT (14 groups; ES = 1.16, 95% CI = 0.54 to 1.79; *I*^2^ = 81.7%; between-moderators categories *p* value = 0.043).

According to the GRADE assessment (Table 6), for in-water vertical jump, in-water agility, time-trial performance, and squat jump, the certainty of evidence is considered low. For horizontal jump, countermovement jump, and body mass, the certainty of evidence is deemed very low. For maximal strength, the certainty of evidence is judged as moderate.

**Table 6:** Certainty of evidence for meta-analysed outcomes

Outcome	No. trials (no. participants)	Comparisons	Certainty of evidence
In-water vertical jump	8 (*n* = 126)	PJT versus specific-active (3 groups) or active controls (1 group)	Low^d^
In-water agility	7 (*n* = 105)	PJT versus specific-active controls	Low^d^
In-water time trials	22 (*n* = 438)	PJT versus specific-active (8 groups) or active controls (9 groups)	Low^a,c^
Maximal strength	5 (*n* = 97)	PJT versus specific-active (3 groups) and active controls (1 group)	Moderate^c^
Horizontal jump	4 (*n* = 105)	PJT versus active controls	Very low^a,b,c^
Squat jump	5 (*n* = 91)	PJT versus specific-active (1 group) and active controls (4 groups)	Low^a,c^
Countermovement jump	20 (*n* = 378)	PJT versus specific-active (8 groups) and active controls (7 groups)	Very low^a,b,c^
Body mass	7 (*n* = 158)	PJT versus specific-active (1 group) and active controls (6 groups)	Very low^a,d^

*PJT* plyometric jump training

^a^Downgraded by one level due to average PEDro score being moderate (< 6)

^b^Downgraded by one level due to high impact of statistical heterogeneity (> 75%)

^c^Downgraded by one level due to < 800 participants for the comparison or unclear direction of the effects

^d^Downgraded by two levels if both < 800 participants for the comparison and unclear direction of the effects were identified

## Discussion

Twenty-six moderate-to-high-quality studies were meta-analysed, involving 618 WSA of both sexes, aged between 10 and 26 years, and from different sports disciplines such as swimming, triathlon, rowing, artistic swimming, and water polo. The results of the meta-analyses showed that PJT interventions induced moderate-to-large improvements (ES = 0.67 to 1.47) in athletes' PF (1RM back squat, horizontal jump distance, squat jump height, and countermovement jump height), and a small improvement in SSP (i.e. time trial speed). Such improvements were noted even when comparison entailed specific-active control groups. Mostly, the level of heterogeneity in the above-mentioned results was low-to-moderate.

Regarding in-water vertical jump height and in-water agility speed (i.e. SSP measures), these did not improve after PJT when compared to control conditions (ES = 0.03 to − 0.15, respectively). Of note, most (for vertical jump) or all (for agility) of the control conditions involved specific-active control groups, meaning that the effect of PJT was compared to an analogous training intervention. Therefore, PJT seems at least equally effective as other training strategies, such as water-specific resistance training, for improving SSP in WSA. Additionally, data regarding in-water vertical jump and agility performance came from one single research group [112, 113, 134, 142], and although the results involved different experiments, all the aforementioned studies included highly trained-professional water polo athletes, meaning that their potential for adaptation is reduced compared with athletes of lower competitive level, or from different sports. Therefore, both in-water vertical jump and in-water agility may have greater chances of improvement following PJT in WSA training at non-professional levels and/or when the effects of PJT are compared to active controls performing their habitual training schedule. Nonetheless, according to GRADE, there is low confidence in these results given that the certainty of evidence for these two outcomes was rated as low. Therefore, future high-quality studies are needed to achieve more conclusive evidence, including WSA with different training experience (e.g. international vs. regional) and water sports of different competitive demands (e.g. mostly metabolic vs. mostly neuromuscular).

With respect to time trial performance tests, these involved swimming distances from 5 m up to 200 m, and rowing distances from 500 m up to 2500 m. Time trial performance improved in WSA after PJT compared to control conditions, involving eight specific-active control groups and nine active controls performing their habitual training schedule. The significant transference effect between PJT exercises and sport-specific performance in other sports such as soccer (e.g. kicking velocity) was previously reported [143–145]. Our analyses additionally suggest that land-based (i.e. isoinertial) PJT has a meaningful transference potential into water-based (i.e. isokinetic) muscle actions. Of note, the transferability of findings from individual PJT studies into WSA practice has been obscured given that most published individual studies have included relatively small sample sizes. Although the results of our meta-analysis contribute to solving previous limitations (i.e. reduced sample size), helping with evidence-based informed decisions regarding PJT implementation [69], it is recommended that future studies in this field include larger sample sizes, provide a full depiction of relevant characteristics of the participants (e.g. previous experience with PJT) and the PJT intervention (e.g. intensity). Such methodological research advances in the field may contribute to improving the confidence in current results.

Improvements in time-trial performance may be related to different physiological adaptations induced after PJT, such as increased motor unit firing rate, improved inter-muscular coordination, improved lower-limb stiffness, increased muscle fibre contraction velocity, and power and force generation capability [29, 146–149]. Such adaptations may transfer to kinetic and kinematic improvements such as greater rate of force development and maximal force generation capability [29, 37, 146–148]. These same physiological adaptations could also explain the increased maximal dynamic strength performance (i.e. 1RM back squat), horizontal jumping performance, and vertical jumping performance noted after PJT compared to control conditions (ES = 0.79 to 1.47). Relatedly, improved dynamic maximal strength and jumping performance may provide significant support to the time-trial performance enhancement noted for WSA. Indeed, up to large associations (*r* = 0.40 to 0.70) have been reported between 20- and 50-m front crawl swimming performance and mean propulsive power in jump squat [16], leg extension strength [17], and horizontal jump distance [18]. Moreover, maximal-intensity short-duration efforts in WSA competitions (e.g. swimmers’ start platform jump; rowers’ stroke) may be a key element of success [150, 151]. Lower-limb fast-force production capabilities (e.g. jumping) may aid performance of such efforts [152–154], for example, increasing the distance per stroke-kick [54, 129, 130]. Improved jumping ability may be associated with an enhanced in-water kicking propulsive force generation capacity [155]. In some WSA (e.g. swimmers), the high- and maximal-intensity short-duration movements (e.g. jump-start; flip turns) represent one-third of the total time-trial performance [156]. This may suggest a meaningful relevance for training interventions aimed at improving WSA high- and maximal-intensity short-duration neuromuscular performance, with PJT offering several advantages to achieve this aim.

Body composition may have a significant impact on WSA performance [8, 22, 23]. Although PJT may induce adaptations in body composition [30, 32], our meta-analyses revealed no effect of PJT compared to control conditions on WSA body mass, fat mass, and thigh girth. Increased power generation capabilities (e.g. greater jumping ability) and unchanged body mass may facilitate generating greater relative power (i.e. W/kg^−1^), an important determinant of performance in WSA [7–9]. Therefore, unchanged body mass may have indirectly contributed to improved time-trial performance, by allowing greater relative power during key movements involving maximal-intensity short-duration efforts (e.g. tumbling-turn in swimming). Regarding thigh girth, its unchanged value (compared to control conditions) may suggest a lack of a hypertrophy-related effect derived from PJT. Nonetheless, PJT may have a skeletal muscle hypertrophy effect [30, 32]. However, the studies that provided data for thigh girth in the current meta-analysis involved PJT interventions lasting only 4 up to 8 weeks, which probably was insufficient to detect any hypothetical hypertrophy effect [157]. Regarding body fat, unchanged values may be expected, due to the relatively low amount of total energy expenditure derived from traditional PJT sessions [30, 42, 72]. Nevertheless, the recent literature suggests that some PJT exercise variations (e.g. greater jumping rate, lower inter-repetition, and inter-set rest) may induce considerable cardiorespiratory responses [158, 159], providing potential for long-term body fat reductions.

Participants' sex (i.e. males vs. females), type of sport (i.e. swimmers vs. water polo athletes), programme duration (i.e. < 8 weeks vs. ≥ 8 weeks), total PJT sessions (i.e. < 18 sessions vs. ≥ 18 sessions), and training period (i.e. pre-season vs. in-season) had no significant moderating effects on time-trial performance changes after PJT. Similar findings were noted for CMJ height, as participants’ sex, type of sport, PJT programme duration, and training period had no significant moderating effects on CMJ changes after PJT. On the contrary, greater (*p* = 0.043) CMJ improvements were noted after ≥ 8 weeks of PJT compared to < 8 weeks of PJT. Previous studies [37, 160, 161] noted that the moderating effects of factors such as participants’ sex, type of sport, PJT duration, and total number of sessions can be outcome-specific. The sex of participants seems to affect jump performance changes after PJT, with no effect on strength or sprint performances [37, 160, 161]. The type of sport practiced seems to moderate sprint, but not strength or jump performances after PJT [37, 160, 161]. Total training duration seems to moderate changes in jump and sprint, but not strength [37, 160, 161]. Therefore, while time trial and CMJ height after PJT seem to be improved in WSA irrespective of participants’ sex or type of water sport, with a minimal effective duration of < 8 weeks and 18–22 total sessions, and with long-term intervention approaches probably increasing chances of significant improvements [162, 163], current novel findings should be considered cautiously. Indeed, due to a limited number of studies, moderator analyses for outcomes other than time trial and CMJ height were precluded in our meta-analysis. Regarding the period of the season, making a comparison with previous studies is difficult. This is because studies addressing the effects of isolated PJT interventions on athletes’ PF according to the season’s period are lacking. However, a previous systematic review found that multimodal interventions, particularly those involving jumping exercises among others, were similarly effective for injury prevention in youth team athletes, regardless of the period of the season [164]. Current findings related to WSA recommend regular implementation of PJT during the season, as commonly occurs in real settings in other sports [165–167].

### Limitations

Despite our systematic review with meta-analysis making a novel and significant contribution to the existing literature and highlighting the benefits of PJT to improve measures of PF and SSP components in WSA, there are some limitations that should be mentioned and discussed. Firstly, a reduced number of studies were available for some outcomes such as in-water agility (i.e. three studies). Secondly, a reduced number of participants (median *n* = 11) were included in most studies. Thirdly, the risk of publication bias analysis was precluded (aside from time-trial performance and countermovement jump) as less than 10 studies were available for most comparisons. Fourthly, the descriptive information provided in some studies was sub-optimal. For example, most of the included studies did not report if the WSA had previous systematic experience with PJT. Further, the intensity of PJT interventions was not detailed in many studies and when reported in some, it was only partially described. Overall, all the included studies did not report one or more key descriptive characteristics of PJT intervention. Fifthly, though I^2^ showed low-to-moderate heterogeneity for most comparisons, two exceptions, horizontal jump distance and countermovement jump height, obtained a high heterogeneity (*I*^2^ = 75.9 to 84.9%). A subgroup and sensitivity analysis would be warranted in such cases. However, due to the extension of this work (e.g. ten outcomes being analysed and discussed; additional analyses; methodological quality assessment), these would be better approached in another publication, focused on secondary analysis. Finally and according to GRADE, the certainty of evidence ranged from very low-to-low for most outcomes, reducing the confidence in the presented estimates.

### Practical Applications and Future Lines for Research

Aside from a greater number of studies needed in this field, future studies should conduct a priori sample size power analysis [65] to recruit a sufficient number of participants, therefore increasing the robustness of their statistical power. Moreover, large randomized-controlled trials should be encouraged in future efforts to address the effects of PJT on measures of PF and SSP components in WSA, providing a proper report of key moderator factors of PJT, such as PJT intensity. Indeed, a sub-analysis of the training intensity factor was precluded due to the lack of a standard method to quantify intensity and the wide variety of approaches used across the included studies. In line with this, to the authors’ knowledge, the effects of PJT intensity on WSA adaptations are currently unknown. Moreover, ~ 35% of the studies conducted in WSA did not provide clear details around the intensity of the applied jump drills. Therefore, the high heterogeneity of the methods used to quantify PJT intensity in addition to the high number of studies (35%) that did not sufficiently report on it precluded any further consideration of this training factor. Nonetheless, a discussion related to PJT intensity has been addressed elsewhere [39, 40, 42, 72, 168–170].

The surface type can affect acute and long-term responses to PJT [29, 41, 171]. Only one out of the 26 studies included in our meta-analysis reported a specific water-based PJT intervention, with the remaining studies using a land-based PJT approach. Compared to water-based, land-based PJT induced greater improvements in lower-limb power [172]. Two reviews of the literature suggested that water-based PJT is as effective as land-based PJT to improve sprint, strength, and jump performance [173, 174]. However, the aforementioned reviews did not focus on WSA. Future studies should clarify the effects of water-based versus land-based PJT on PF and SSP outcomes in WSA.

Similar to the surface type, only 4 out of 26 of the included studies in this meta-analyses used a tapering approach. This is considered an important programming variable for PJT [175] and competitive performance [176–178], particularly after interventions involving a large volume-load of training, commonly occurring for WSA [7–11]. Moreover, some PJT interventions involved up to ~ 28,000 total jumps. Future studies may analyse the effects of different tapering strategies on the PF and SSP outcomes in WSA.

In some competitive contexts, a greater emphasis may be provided on PJT. For example in swimmers, greater time-trial improvement may be expected after PJT for distances completed in shorter length pools. Shorter pools would mean that the time-trial distance covered due to the jump-start and flip-turn (i.e. jump-like action) movements represents a greater proportion of the total distance. Such actions are key elements of success in swimming [150, 151] and are expected to improve with PJT, as suggested by current meta-analyses.

## Conclusions

PJT is a more effective method to improve measures of PF and SSP in WSA compared to control conditions involving traditional sport-specific training only as well as alternative training interventions. This conclusion is derived from 26 articles of moderate-to-high methodological quality, low-to-moderate heterogeneity for most outcomes, and very low-to-moderate certainty of evidence according to GRADE.

## Supplementary Information

**Additional file 1.** Search strategy (code line) for each database and background of search history.

**Additional file 2.** Additional exclusion criteria.

**Additional file 3.** Exclusion reasons for studies included in preliminary qualitative synthesis.

## Data Availability

All data generated or analysed during this study are included in the article as Table(s), Figure(s), and/or Electronic Supplementary Material(s). Any other data requirement can be directed to the corresponding author upon reasonable request.

## Abbreviations

CI: Confidence interval
ES: Effect sizes
GRADE: Grading of recommendations assessment, development, and evaluation
VO_2max_: Maximum oxygen consumption
1RM: One repetition maximum
PF: Physical fitness
PEDro: Physiotherapy evidence database
PICOS: Participants, intervention, comparators, outcomes, and study design
PJT: Plyometric jump training
PRISMA: Preferred reporting items for systematic reviews and meta-analyses
SSP: Sport-specific performance
