# Supplementary material for: Effects of Plyometric Jump Training on Measures of Physical Fitness and Sport-Specific Performance of Water Sports Athletes: A Systematic Review with Meta-analysis
Source: Sports Med Open. 2022 Aug 29;8:108. doi: 10.1186/s40798-022-00502-2 (PMC9424421; doi:10.1186/s40798-022-00502-2)
Supplement: Supplementary file 1 — Additional file 1. Search strategy (code line) for each database and background of search history. [file 40798_2022_502_MOESM1_ESM.docx]

**Electronic Supplementary Material Table S1**

**Article title**: Effects of plyometric jump training on measures of physical fitness and sport-specific performance of water sports athletes: a systematic review with meta-analysis

**Author names**: Rodrigo Ramirez-Campillo, Alejandro Perez-Castilla, Rohit K. Thapa, José Afonso, Filipe Manuel Clemente, Juan C. Colado, Eduardo Saéz de Villarreal, Helmi Chaabene

**Affiliation and e-mail of the corresponding author**:

Helmi Chaabene, Ph.D.

Department of Sports and Health Sciences, Faculty of Human Sciences, University of Potsdam, D-14469 Potsdam, Germany. Mail: chaabene@uni-potsdam.de

Table S1. Search strategy (code line) for each database and background of search history.

| **Date of the search** | April, 2017 | May, 2019 | August, 2021 |
| --- | --- | --- | --- |
| **Databases** | PubMed | PubMed, WOS (Core Collection), Scopus | PubMed, WOS (Core Collection) ^a^, Scopus |
| **Keywords** | “plyometric”, “training” | “ballistic”, “complex”, “cycle”, “explosive”, “force”, “plyometric”, “shortening”, “stretch”, “training”, “velocity” | “ballistic”, “complex”, “cycle”, “explosive”, “force”, “jump”, “plyometric”, “power”, “shortening”, “stretch”, “training”, “velocity” |
| **Database fields for the search** | All | PubMed: all  WOS: all  Scopus: title, abstract, keywords | PubMed: all ^b^  WOS: all ^b^  Scopus: title, abstract, keywords ^b^ |
| **Restrictions for the search** | None | None | None |
| **Examples of search strategy code line** | PubMed: "plyometric exercise"[MeSH Terms] OR ("plyometric"[All Fields] AND "exercise"[All Fields]) OR "plyometric exercise"[All Fields] OR ("plyometric"[All Fields] AND "training"[All Fields]) OR "plyometric training"[All Fields]  WOS: (ALL=(plyometric)) AND ALL=(training)  Scopus: TITLE-ABS-KEY ( plyometric AND training ) | | |
| ^a^: except for the keywords “jump” and “power” searched in all WOS databases; ^b^: except for the keywords “jump” and “power” searched in the database field TITLE (a very poor efficiency was obtained in the search for results with the incorporation of other database fields); Note: after formal database search, the list of included articles and the inclusion criteria (see Table 1) were sent to three independent world experts in the field of physical fitness and sport-specific performance, plyometric-jump training, and water sport athlete (<https://www.expertscape.com/ex/physical+fitness>; <https://www.expertscape.com/ex/plyometric+exercise>; https://www.expertscape.com/ex/water+sports) to help identify additional relevant articles. Additionally, the experts (i) held a Ph.D. in Sports Sciences or related field (e.g., Health Sciences), and (ii) have peer-reviewed publications in the fields of physical fitness and sport-specific performance, plyometric-jump training and/or water sport athlete. The experts were not provided with our search strategy, to avoid biasing their own searches. Upon completion of all these steps, the databases were again consulted in search for errata or retractions of any included study. | | | |
