# Supplementary material for: Effects of Plyometric Jump Training on Measures of Physical Fitness and Sport-Specific Performance of Water Sports Athletes: A Systematic Review with Meta-analysis
Source: Sports Med Open. 2022 Aug 29;8:108. doi: 10.1186/s40798-022-00502-2 (PMC9424421; doi:10.1186/s40798-022-00502-2)
Supplement: Supplementary file 2 — Additional file 2. Additional exclusion criteria. [file 40798_2022_502_MOESM2_ESM.docx]

**Electronic Supplementary Material Table S2**

**Article title**: Effects of plyometric jump training on measures of physical fitness and sport-specific performance of water sports athletes: a systematic review with meta-analysis

**Author names**: Rodrigo Ramirez-Campillo, Alejandro Perez-Castilla, Rohit K. Thapa, José Afonso, Filipe Manuel Clemente, Juan C. Colado, Eduardo Saéz de Villarreal, Helmi Chaabene

**Affiliation and e-mail of the corresponding author**:

Helmi Chaabene, Ph.D.

Department of Sports and Health Sciences, Faculty of Human Sciences, University of Potsdam, D-14469 Potsdam, Germany. Mail: chaabene@uni-potsdam.de

Table S2. Additional exclusion criteria.

| Excluded were books, book chapters, and congress abstracts, as well as cross-sectional and review papers, and training-related studies that did not focus on the effects of plyometric jump training (PJT) exercises, such as plyometric training without the use jumps (e.g., upper-body plyometrics only). Also excluded were retrospective studies, prospective studies (e.g., relationship between bone density at the end of PJT, and at several years of follow-up), studies in which the use of PJT exercises was not clearly described (e.g., authors stated “plyometric exercises were implemented”, without further explanation), studies for which only the abstract was available, case reports, special communications, letters to the editor, invited commentaries, errata, studies with doubtful quality or unclear peer-review process from the journal [1], overtraining studies, and detraining studies. In the case of detraining studies, these were considered for inclusion if involved a training period prior to a detraining period. |
| --- |

1. Grudniewicz A, Moher D, Cobey KD, Bryson GL, Cukier S, Allen K, et al. Predatory journals: no definition, no defence. Nature. 2019 Dec;576(7786):210-2.
