# Supplementary material for: Effects of Plyometric Jump Training on Measures of Physical Fitness and Sport-Specific Performance of Water Sports Athletes: A Systematic Review with Meta-analysis
Source: Sports Med Open. 2022 Aug 29;8:108. doi: 10.1186/s40798-022-00502-2 (PMC9424421; doi:10.1186/s40798-022-00502-2)
Supplement: Supplementary file 3 — Additional file 3. Exclusion reasons for studies included in preliminary qualitative synthesis. [file 40798_2022_502_MOESM3_ESM.docx]

**Electronic Supplementary Material Table S3.**

**Article title**: Effects of plyometric jump training on measures of physical fitness and sport-specific performance of water sports athletes: a systematic review with meta-analysis

**Author names**: Rodrigo Ramirez-Campillo, Alejandro Perez-Castilla, Rohit K. Thapa, José Afonso, Filipe Manuel Clemente, Juan C. Colado, Eduardo Saéz de Villarreal, Helmi Chaabene

**Affiliation and e-mail of the corresponding author**:

Helmi Chaabene, Ph.D.

Department of Sports and Health Sciences, Faculty of Human Sciences, University of Potsdam, D-14469 Potsdam, Germany. Mail: chaabene@uni-potsdam.de

**Table S3.**

Exclusion reasons for studies included in preliminary qualitative synthesis.

| **Article** | **Reason** |
| --- | --- |
| Escriva-Selles and Gonzalez-Badillo [1] | Comparator-related reasons (e.g., inappropriate control group). Although studies comparing different plyometric-jump training approaches without active or traditional control group were considered, this was the only study to include a loaded training group and an unloaded group, precluding meta-analysis. |
| Kamandulis et al. 2012 [2] | Participant-related reasons (e.g., mixed sports). The authors mixed basketball players, swimmers, and endurance runners. |
| Lyttle et al. 1996 [3] | Participant-related reasons (e.g., mixed sports). The authors mixed track and field athletes, rugby players, and swimmers. |
| Marques et al. 2020 [4] | Comparator-related reasons (e.g., inappropriate control group). Although studies comparing different plyometric-jump training approaches without active or traditional control group were considered, this was the only study to include a female training group and a male group, precluding meta-analysis. |
| Polhemus and Burkhardt 1980 [5] | Participant-related reasons (e.g., mixed sports). The authors mixed track and field athletes, basketball players, and swimmers. |
| Rejman et al. 2017 [6] | Comparator-related reasons. No control group. |
| Van der Zwaard et al., 2021 [7] | Comparator-related reasons. This study used an observational design, involving 19 athletes monitored during their general preparation period of the training season, and then involving 7 athletes during the competitive preparation period, thus, precluding an adequate control condition. |

**REFERENCES**

1. Escrivá-Sellés FR, González-Badillo JJ. Effect of two periods of power training on performance in the thrust, barracuda and boost exercises in synchronised swimming. Apunts Educación Física y Deportes. 2020;142:35-45.

2. Kamandulis S, Snieckus A, Venckunas T, Aagaard P, Masiulis N, Skurvydas A. Rapid increase in training load affects markers of skeletal muscle damage and mechanical performance. J Strength Cond Res. 2012;26(11):2953-61.

3. Lyttle AD, Wilson GJ, Ostrowski KJ. Enhancing performance: maximal power versus combined weights and plyometrics training. J Strength Cond Res. 1996;10(3):173-9.

4. Marques MC, Yáñez-García JM, Marinho DA, González-Badillo JJ, Rodríguez-Rosell D. In-season strength training in elite junior swimmers: the role of the low-volume, high-velocity training on swimming performance J Human Kinetics. 2020;74(1):71-84.

5. Polhemus R, Burkhardt E. The effects of plyometric training with ankle and vest weights on conventional weight training programs for mess and women. National Strength Coaches Association Journal. 1980;2(1):13-5.

6. Rejman M, Bilewski M, Szczepan S, Klarowicz A, Rudnik D, Mackala K. Assessing the impact of a targeted plyometric training on changes in selected kinematic parameters of the swimming start. Acta of bioengineering and biomechanics / Wroclaw University of Technology. 2017;19(2):149-60.

7. van der Zwaard S, Koppens TFP, Weide G, Levels K, Hofmijster MJ, de Koning JJ, et al. Training-induced muscle adaptations during competitive preparation in elite female rowers. Frontiers in sports and active living. 2021;3.
